# Grapevine woody tissues accumulate stilbenoids following bud burst

**DOI:** 10.1007/s00425-023-04270-5

**Published:** 2023-11-14

**Authors:** Henrique Noronha, Angélica Silva, Virginie Garcia, Kévin Billet, Alberto C. P. Dias, Arnaud Lanoue, Philippe Gallusci, Hernâni Gerós

**Affiliations:** 1 Department of Biology, Centre of Molecular and Environmental Biology (CBMA), University of Minho, Braga, Portugal; 2 UMR EGFV, Bordeaux Sciences Agro, INRAE, Université de Bordeaux, 210 Chemin de Leysotte, CS 50008, 33882 Villenave d’Ornon, France; 3 EA 2106 Biomolécules et Biotechnologies Végétales, UFR des Sciences Pharmaceutiques, Université de Tours, 31 Av. Monge, 37200 Tours, France

**Keywords:** Phenylpropanoid pathway, Resveratrol, Shikimate pathway, Viniferin, *Vitis vinifera*, Woody tissues

## Abstract

**Main conclusion:**

After bud burst, a transcriptional reprogramming of the shikimate and phenylpropanoid pathways occurs in grapevine canes resulting in the accumulation of stilbenoids like resveratrol and viniferin.

**Abstract:**

Stilbenoids are phenylpropanoid compounds with important biological properties and biotechnological applications that are synthesized in grapevine in response to different stresses. Although they are found in woody tissues, such as canes and buds, their biosynthesis and accumulation have been essentially described in berries. We have previously shown that transcripts encoding secondary metabolism enzymes accumulate in grapevine canes following the transition from dormancy (E-L 1) to bud burst (E-L 4) suggesting that secondary metabolites may accumulate in grapevine canes during this transition. In the present study, using UPLC-MS we demonstrate the accumulation of important metabolites such as ferulic acid and the stilbenoids *E-*resveratrol, *E-*piceatannol and *E-*ε-viniferin. Stilbenoids accumulation correlated with the increased expression of several *stilbene synthase* genes and of *VviMYB14*, encoding a transcription factor that regulates stilbene biosynthesis. In addition, a general stimulation of the plastidial shikimate pathway was observed. Taken together, results show that important secondary metabolites accumulate in the woody canes during bud burst. These findings may aid biotechnological approaches aimed at extracting biologically active phenolic compounds, including stilbenoids, from grapevine woody tissues.

**Supplementary Information:**

The online version contains supplementary material available at 10.1007/s00425-023-04270-5.

## Introduction

**Fig. 1:**
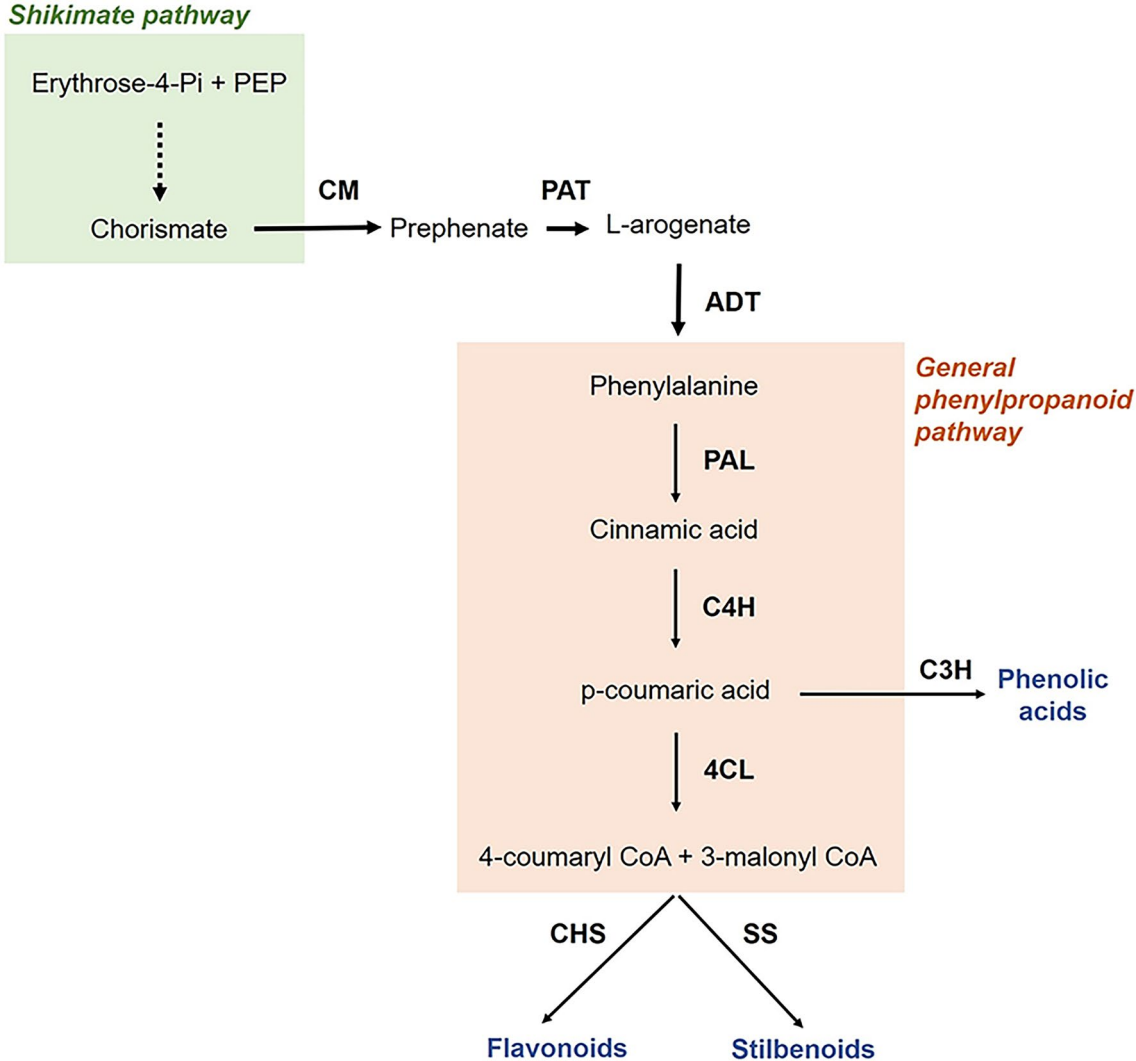
Simplified representation of the shikimate and phenylpropanoid pathways and their connection via the aromatic amino acid phenylalanine. *CM* chorismate mutase, *PAT* prephenate aminotransferase, *ADT* arogenate dehydratase, *PAL* phenylalanine ammonia lyase, *C4H* cinnamate 4-hydroxylase, *4CL* 4-coumarate-CoA ligase, *C3H* coumarate 3-hydroxylase, *CHS* chalcone synthase, *SS* stilbene synthase

The grapevine secondary metabolism has been extensively studied, with a focus on the phenylpropanoid pathway due to its importance in fruit and wine quality (Matus 2016). Phenylpropanoid biosynthesis starts from the aromatic amino acid phenylalanine, which is produced in plastids by the shikimate pathway. Flavonoids, stilbenoids, and phenolic acids are subsequently generated from intermediates of the phenylpropanoid pathway through the action of specific enzymes (Fig. 1; Vogt 2010), including chalcone and stilbene synthases which play a fundamental role in directing the carbon flux into flavonoids or stilbenoids synthesis depending on environmental, hormonal, and biotic cues (Dao et al. 2011). Stilbenoids, especially resveratrol, are among the most studied plant secondary metabolites due to their recognized human health benefits. They possess potent antioxidant, antibacterial, antifungal, cardioprotective, neuroprotective, antiaging, and anticancer properties (Biais et al. 2017).

The grapevine secondary metabolism has been mainly studied in grape berries and leaves, and *E*-resveratrol has been shown to accumulate in the exocarp, seeds and infected leaves (Gatto et al. 2008; Németh et al. 2017; Billet et al. 2020). However, *E*-resveratrol and other stilbenoids, such as *E*-piceid and *E*-ε-viniferin, are known to accumulate at much higher levels in perennial tissues, including canes and roots and in winter buds (Németh et al. 2017). Additionally, different stilbenoids like ampelopsin A, *E*-miyabenol C, *Z*/*E*-vitisin B, hopeaphenol, and isohopeaphenol are also accumulated in grapevine canes (Lambert et al. 2013; Houillé et al. 2015a, 2015b). Stilbenoids accumulation in canes is genotype dependent (Lambert et al. 2013; Billet et al. 2021), and in winter-harvested canes its accumulation is modulated by storage temperatures (Houillé et al. 2015b), while downy mildew infections stimulate stilbenoids synthesis during the growing season (Houillé et al. 2015a).

The induction and release of dormancy in perennial woody fruit crops, for which grapevine (*Vitis vinifera* L.) stands as a model, is finely tuned by complex environmental and endogenous signals. Although the physiological modifications that occur in the buds have been well-characterized (Zheng et al. 2015), the understanding of processes initiated in woody tissues upon dormancy release is still limited (Noronha et al. 2018). In a recent transcriptomic analysis, we have shown that genes involved in secondary metabolic pathways, including those of the phenylpropanoid pathway, were highly upregulated in woody tissues located below the bud after inducing bud burst (Noronha et al. 2021). Here, we demonstrate that the transcriptional activation of these genes results in the accumulation of important metabolites including ferulic acid and the stilbenoids *E-*resveratrol, *E-*piceatannol and *E*-ε-viniferin. We analyzed the expression of genes encoding enzymes of the shikimate and stilbene pathways. Overall, results are consistent with a general stimulation of the different pathways necessary for phenylpropanoid synthesis, which suggests that during bud burst carbon is being fueled to the phenylpropanoid pathway.

## Material and methods

### Plant material

Bud burst was induced following the protocol established by Noronha et al. (2021; Supplementary Fig. S1). Briefly, lignified grapevine canes from cv. Vinhão were collected from a commercial vineyard of the Controlled Appellation (DOC) region of Vinhos Verdes in the northwest region of Portugal (41°48′45.3″N 8°24′36.4″W) in 2018 and stored at 4 °C until the beginning of the experiment. Shoots of similar diameter were chosen and cut into single bud segments of approximately 15 cm and placed in the growth chamber (23 °C, 12/12 h photoperiod) in wet floral foam. When samples reached the E-L 4 stage, 3 cm segments just below the bud were cut, frozen in liquid N_2_, ground in an IKA A11 basic analytical mill, and stored at -80 ºC. Control samples were collected just before the beginning of the experiment (dormancy; E-L 1). At each sampling point, 3 or 4 independent cane segments were collected and pooled in 3 groups (biological replicates). The modified E-L system was used to classify the bud developmental stages (Pearce and Coombe 2004).

### RNAseq analysis

Data treatment was previously performed on Noronha et al. (2021). Briefly, genes assigned as differentially expressed (*P* value < 0.05 and |log2FC|> 1.0) by DESeq (Anders and Huber 2010) were categorized according to the MapMan ontology (× 4.1) with Mercator tool (Lohse et al. 2014) and MapMan standalone software (v3.5.1) was used to explore the data.

### Metabolomic analysis UPLC-MS

After lyophilization of ground samples, 50 mg were extracted in 1 mL ethanol/water solution (60:40; v/v), shaken for 30 min at 1400 rpm at 83 °C and centrifuged at 18 000 g for 5 min. The extracts were diluted 1:5 with 0.1% formic acid and stored at − 20 °C until analyses. UPLC-MS was performed using an ACQUITY™ Ultra Performance Liquid Chromatography system coupled to a photodiode array detector (PDA) and a Xevo TQD mass spectrometer (Waters, Milford, MA, USA) equipped with an electrospray ionization (ESI) source controlled by Masslynx 4.1 software (Waters). Analyte separation was achieved by using a Waters Acquity HSS T3 C18 column (150 mm × 2.1 mm, 1.8 µm) with a flow rate of 0.4 mL min^−1^ at 55 °C. The injection volume was 5 µL. The mobile phase consisted of solvent A (0.1% formic acid in water) and solvent B (0.1% formic acid in acetonitrile). Chromatographic separation was achieved using an 18-min linear gradient from 5 to 60% solvent B. MS detection was performed in both positive and negative modes. The capillary voltage was 3000 V and sample cone voltages were 30 and 60 V. The cone and desolvation gas flow rates were 60 and 800 L h^−1^. Identification of analytes was based on retention times, *m*/*z* values, and UV spectra as previously described (Billet et al. 2018a, b). Absolute quantifications were conducted using pure standards of tyrosine, tryptophan, phenylalanine, catechin, epicatechin, procyanidin B1, procyanidin B2, procyanidin B3, procyanidin B4, gallic acid, caffeic acid, ferulic acid, coutaric acid, caftaric acid, *E*-resveratrol, *E*-piceatannol, *E*-ε-viniferin, *E*-miyabenol C, ampelopsin A, *Z*/*E*-vitisin B and hopeaphenol using a five-point calibration curve (0–10 ppm). *Z*-ϵ-viniferin, ω-vinferin, δ-viniferin were expressed as *E*-ε-viniferin equivalent.

### Analysis by HPLC–DAD

Methanolic extracts were prepared as indicated above. Samples were run at 0.8 mL/min, using 0.1% formic acid in water (eluent A) and 0.1% formic acid in methanol (eluent B) as mobile phase, through a Purospher Star RP-18 (25 × 0.4 mm, particle size of 5 µm, Merck) column connected to an HPLC (Hitachi—LaChrom Elite) equipped with a DAD (Diode Array Detector). The elution gradient start with 5% eluent B at 0 min to 95% B after 45 min. Detection was acquired in the range of 230–650 nm for obtention of compounds UV–Vis spectra. Principal Component Analysis Biplot (PCA) was performed in R Studio software version 4.1.0 using the FactoMineR package v1.34 (Lê et al. 2008).

### Quantification of total phenolics and antioxidant activity in methanolic extracts

Phenolic compounds were extracted from 100 mg of lyophilized cane tissues by adding 1 mL of 80% methanol, mixing thoroughly, and an overnight incubation at 4 °C. Following this, the mixture was centrifuged, and the supernatants filtered. Total phenolics were quantified using the Folin-Ciocalteu method. Briefly, 10 µL of methanolic extracts were mixed with 100 µL of Folin reagent and 1.58 mL of H_2_O and, after 5 min incubation, 300 µL of 3 M sodium carbonate. After 2 h incubation in the dark, absorbance was measured at 765 nm and gallic acid was used as standard. Antioxidant capacity was assessed using the 2,2-diphenyl-1-picrylhydrazyl (DPPH) method. Briefly, 10 µL of methanolic extracts were incubated with 140 µL of 400 µM DPPH, the reaction incubated for 30 min, and the absorbance measured at 517 nm. Quercetin was used as standard.

### Phenylalanine ammonia-lyase (PAL) activity

Phenylalanine ammonia-lyase (PAL) activity was determined using the protocol of Conde et al. (2016). Total protein extracts were obtained by mixing approximately 200 mg of from cane tissues with 1 mL of extraction buffer [50 mM Tris–HCl pH 8.9, 5 mM MgCl_2_, 1 mM EDTA, 1 mM phenylmethylsulfonyl fluoride, 5 mM dithiothreitol, and 0.1% (v/v) Triton X-100] followed by thorough mixing. The homogenates were centrifuged for 20 min at 18,000 g and the supernatants were collected and maintained on ice until the PAL enzymatic determination. Total protein concentrations of the extracts were determined by the Bradford method using bovine serum albumin as a standard (Bradford 1976). PAL enzymatic activity was determined in crude cane protein extracts by continuously monitoring the conversion of L-phenylalanine to trans-cinnamic acid at 290 nm. The 2 mL reaction mixture contained 0.2 mL of enzyme extract, 3.6 mM NaCl, and 25 mM L-phenylalanine (a saturating concentration) as substrate in 50 mM Tris–HCl, pH 8.9. Enzyme activity was calculated using the trans-cinnamic extinction coefficient of 16,890 M cm^−1^.

### Real-time PCR studies

Total RNA from cane tissues was isolated as previously described by Reid et al. (2006) following some adaptations (Noronha et al. 2021). For each condition, 500 mg of frozen tissue was mixed with 1 mL of extraction buffer containing 300 mm Tris HCl (pH 8.0), 25 mm EDTA, 2.0 M NaCl, 2% CTAB, 2% PVP (K-30), and 30 mm DTT. Samples were then incubated at 60 °C for 15 min and shaken every 5 min. Then, mixtures were extracted twice with 850 μL of chloroform: isoamyl alcohol (24:1, v/v) followed by centrifugation at 15,000 g for 15 min at 4 °C. Subsequently, 0.1 vol of 3 M NaOAc (pH 5.2) and 0.6 vol of isopropanol were added to the aqueous phase, followed by incubation at − 80 °C for 1 h. The mixture was centrifuged at 15,000*g* at 4 °C for 30 min and resuspended in 100 μL of H_2_O. To purify the samples, the GRS Total RNA Kit—Plant (GriSP, Lda., Porto, Portugal) was used following the manufacturer’s instructions. RNA concentration and purity were quantified spectrophotometrically in a NanoDrop ND-1000 (Termo Fisher Scientifc Inc.) and integrity checked in a 1% (w/v) agarose gel. First-strand cDNA synthesis was performed using the Xpert cDNA Synthesis Mastermix protocol (GriSP, Lda.).

Quantitative real-time PCRs were performed with Xpert Fast SYBR Blue (GriSP, Lda.) along with the conditions previously optimized in a CBX96 Real-Time Detection System (Bio-Rad). The amplification protocol included an initial denaturation step at 95 °C for 3 min, followed by an additional 40 cycles of denaturation for 3 s at 95 °C and 30 s at 60 °C. Experiments were done in biological replicates and then interpreted with the Bio-Rad CFX Manager (Bio-Rad) software, while *VviGAPDH* (glyceraldehyde-3-phosphate dehydrogenase) and *VviACT1* (actin) were used as internal control. The primers used in this study are listed in Supplementary Table S1.

### Statistical analysis

Results were analyzed in the Prism vs. 6 (GraphPad Software, Inc.) using Analysis of Variances tests (oneway ANOVA). Statistical differences between samples were marked with asterisks (*P ≤ 0.05; ***P < 0.001; ****P < 0.0001).

## Results

### Transcriptomic analysis of phenylpropanoid and shikimate pathways in grapevine woody tissues following bud burst

**Fig. 2:**
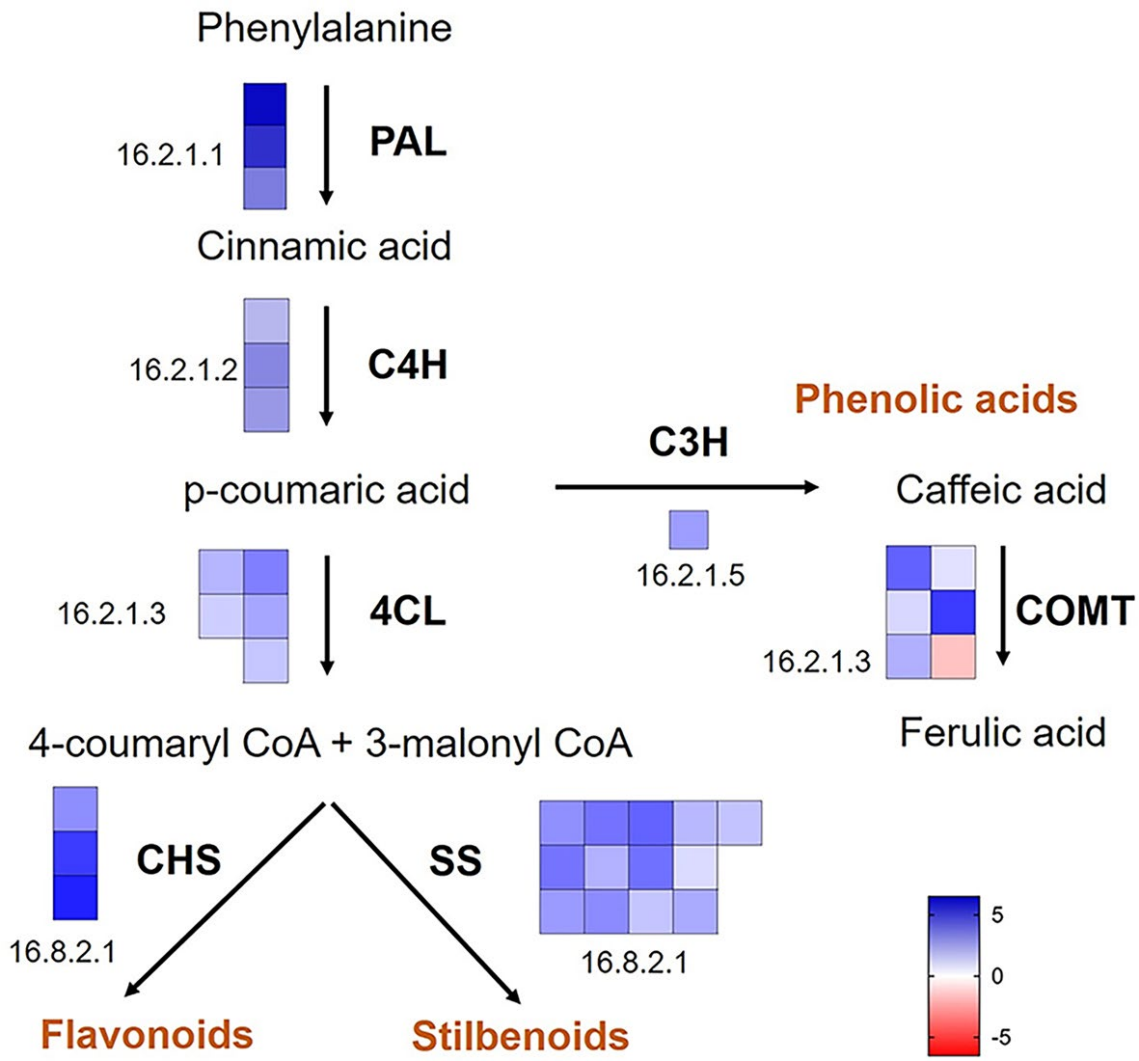
MapMan pathway for phenylpropanoid pathway with DEGs. Each square represents a gene. In blue, genes more expressed at E-L 4 (bud burst); in red, genes more expressed at E-L 1 (dormancy). Bin numbers can be found in additional files with gene details. *PAL* phenylalanine ammonia lyase, *C4H* cinnamate 4-hydroxylase, *4CL* 4-coumarate-CoA ligase, *C3H* coumarate 3-hydroxylase, *COMT* catechol-O-methyltransferase, *4CL* 4-coumarate-CoA ligase, *CHS* chalcone synthase, *SS* stilbene synthase

The transcriptome profile was analyzed in woody tissues before (E-L 1) and after bud burst (E-L 4) using previously generated RNAseq data (Noronha et al. 2021). The visualization of the phenylpropanoid pathway using MapMan showed a general stimulation of differentially expressed transcripts at E-L 4 (Fig. 2). Particularly, genes encoding three PAL (bin 16.2.1.1), the first enzyme of the phenylpropanoid pathway, and those coding for 13 stilbene synthases and 3 chalcone synthases (bin 16.8.2.1) were upregulated by up to fivefold (log2 fold expression). In grapevine, 48 putative *VviSTS* gene sequences were identified, and at least 31 of them encode full-length proteins (Ciaffi et al. 2019). To our knowledge, it has been shown that *VviSTS4* (named *Vst1*; Hain et al 1993), *VviSTS15* and *VviSTS22* (named *vst1* and *vst2*; Thomzik et al. 1997) and *VviSTS2*, *-5*, *-7*, *-10*, *-16*, *-20*, *-21*, *-25* and *-29* (Parage et al. 2012) are able to synthesize stilbenes. Due to their high sequence similarity, transcript validation by qPCR was limited because genes are highly homologous, and most of them cannot be analyzed separately (Ciaffi et al. 2019)*.* However, results confirmed that *VviSTS3/4*, *-7/8*, *-14* and *-20* were upregulated in woody tissues at E-L 4 (Supplementary Fig. S2). In addition, *VviMYB14*, which codes for a transcription factor involved in the regulation of stilbene synthesis (Höll et al. 2013), was upregulated threefold from E-L 1 to E-L 4, while the expression of *VviMYB15* was not detected by qPCR (Supplementary Fig. S2).

**Fig. 3:**
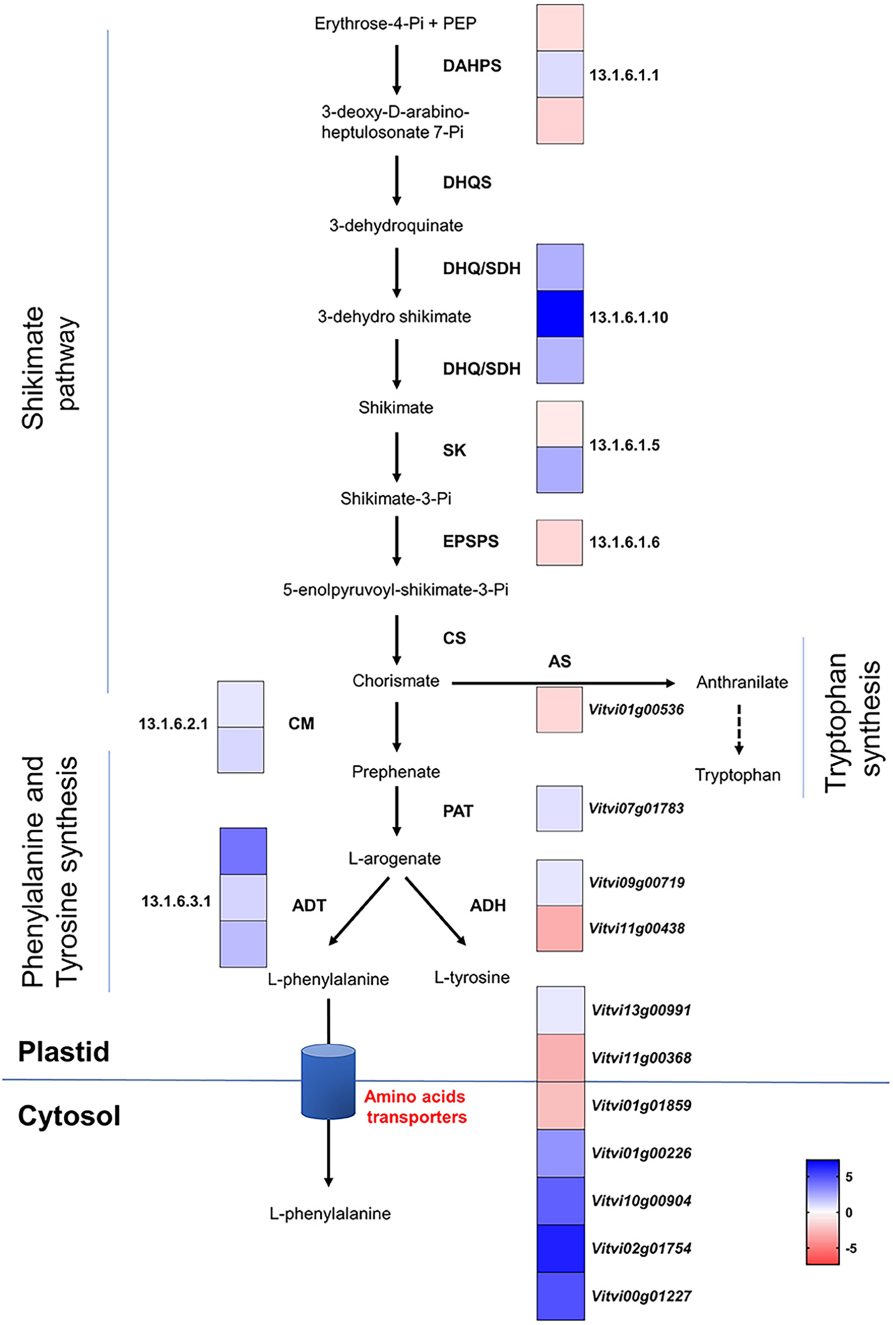
MapMan pathway for shikimate pathway with DEGs. Each square represents a gene. In blue, genes more expressed at E-L 4 (bud burst); in red, genes more expressed at E-L 1 (dormancy). When MapMan bin not available, grapevine VCost.v3 accession numbers are shown. Bin numbers can be found in additional files with gene details. *DAHPS* 3-deoxy-d-arabinoheptulosonate 7-phosphate synthase, *DHQS* 3-dehydroquinate synthase, *SDH* shikimate dehydrogenase, *SK* shikimate kinase, *EPSPS* 5-enolpyruvylshikimate-3-phosphate synthase, *CM* chorismate synthase, *CM* chorismate mutase, *AS* anthranilate synthase, *PAT* prephenate aminotransferase, *ADT* arogenate dehydratase, *ADH* arogenate dehydrogenase

RNA seq analysis also shows an increase in the transcript levels of key genes of the shikimate pathway when comparing E-L 1 to E-L 4 (Fig. 3). This is true for *DAHPs* (3-deoxy-d-arabinoheptulosonate 7-phosphate synthase) (except *VviDAHPS3*), which codes the first enzyme of the shikimate pathway, and for the *ADT* gene encoding arogenate dehydratases, which convert arogenate into phenylalanine. Also, the expression of several plastidial aromatic amino acid transporters was upregulated at E-L 4, namely *VviCAT6* (Vitvi02g01754). (Fig. 3).

### Metabolomic analysis of grapevine canes by HPLC–DAD and UPLC-MS

**Fig. 4:**
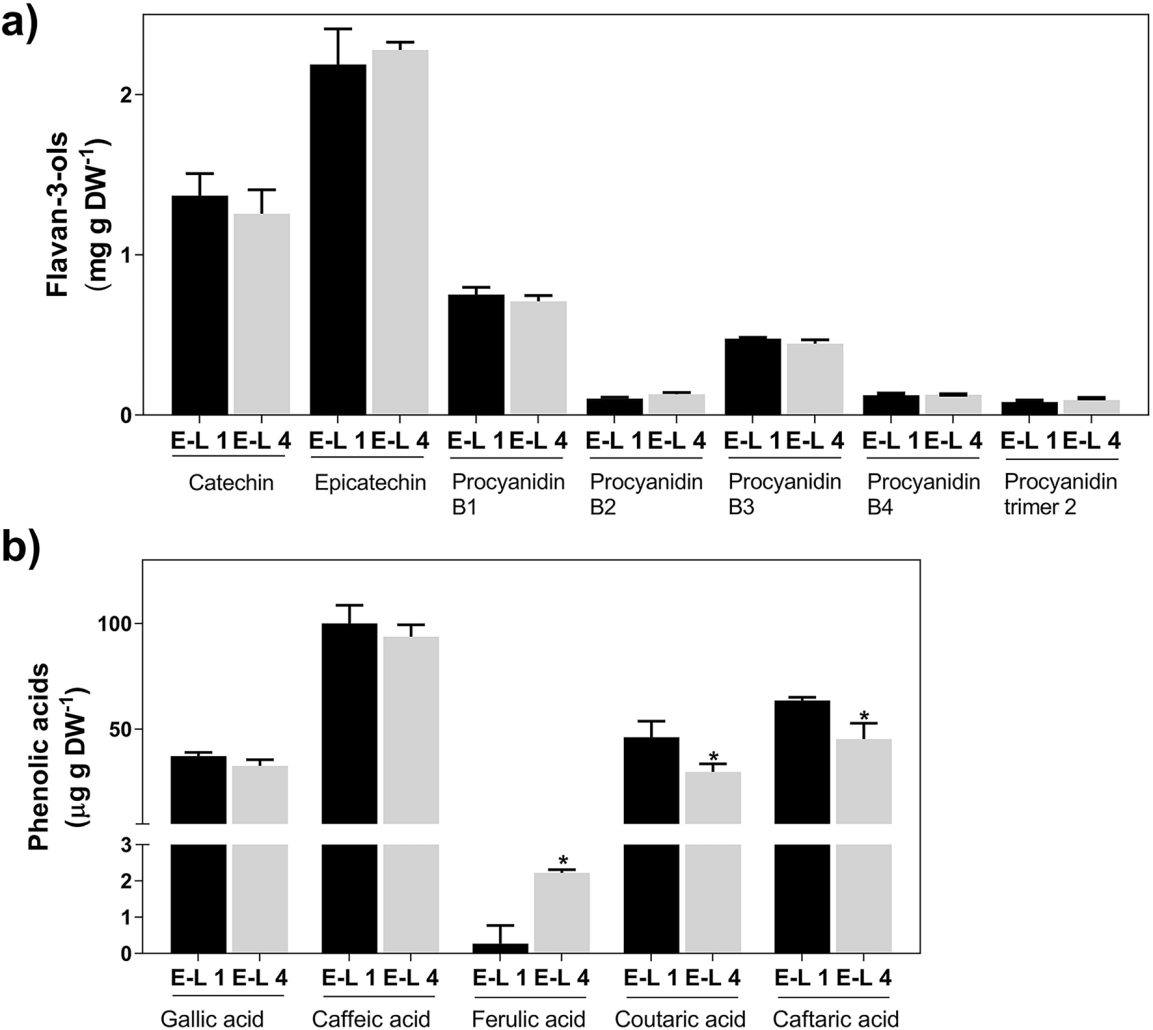
Metabolomic analysis of flavan-3-ols (**a**) and phenolic acids (**b**) in grapevine canes at E-L 1 and E-L 4. Asterisks denote statistically significant differences between E-L 4 and E-L 1 (Mean values ± SD, *n* = 3, *P ≤ 0.05)

The PCA of the HPLC–DAD analysis showed a clear separation between phenolic extracts from E-L 1 and E-L 4 (3-cm woody canes below the emerging bud), with PC1 accounting for 63% of the variation and PC2 for 16% (Supplementary Fig. S3). The targeted metabolomic analysis by UPLC-MS performed in the present study identified 25 metabolites, including phenolic acids, flavan-3-ols, and stilbenoids. Figure 4 shows the profile of variation of phenolic acids and flavan-3-ols from E-L 1 to E-L 4. While no changes were observed in the amounts of flavan-3-ols (Fig. 4a), some key phenolic acids changed following the bud burst. This is the case of ferulic acid that increased tenfold after bud burst (from 0.2 at E-L 1 to 2.2 µg g DW^−1^ at E-L 4; Fig. 4b). Conversely, coutaric and caftaric acids levels in woody tissues were reduced by up to 35% from E-L 1 to E-L 4.

**Fig. 5:**
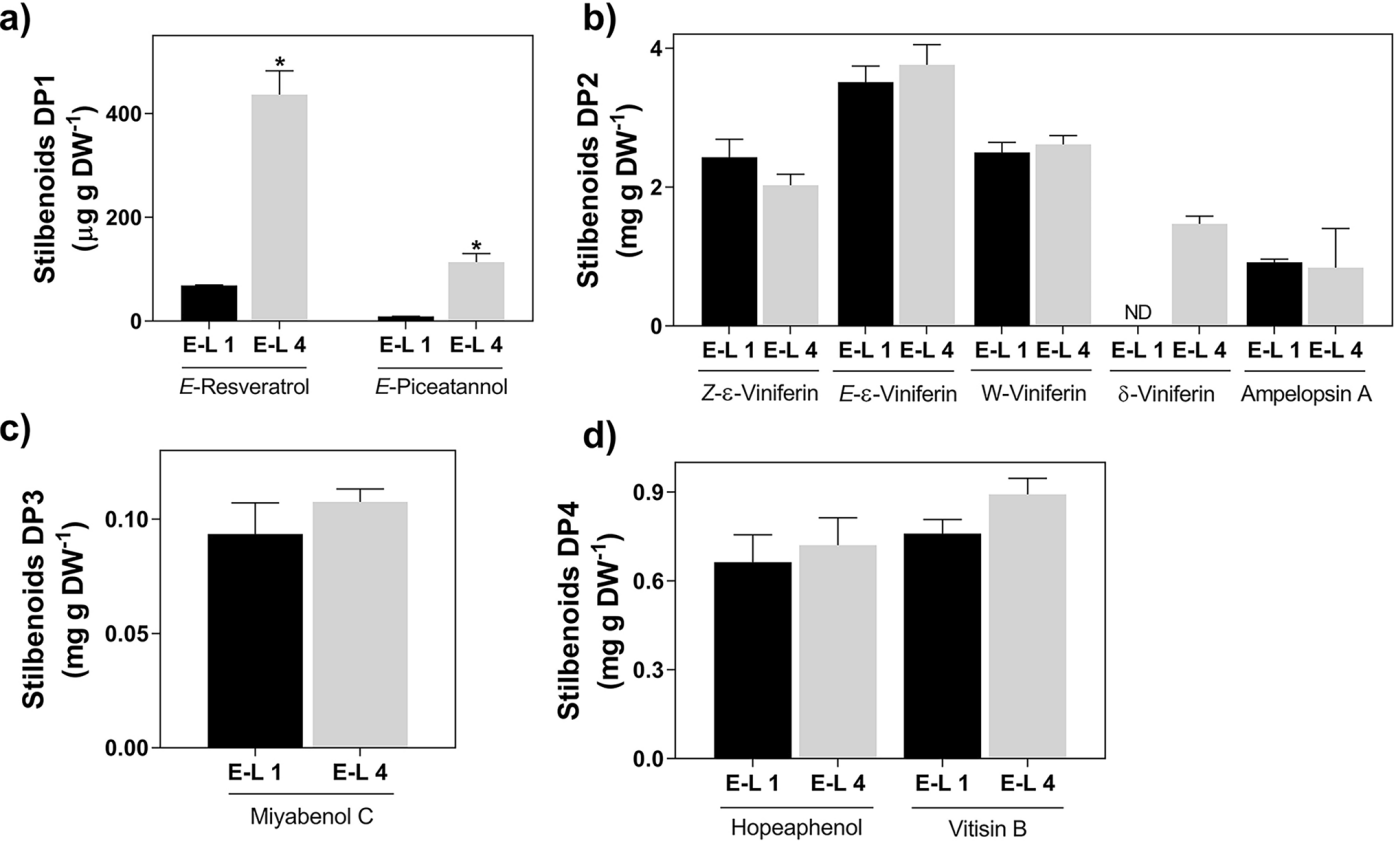
Metabolomic analysis of stilbenoids DP1 (**a**), DP2 (**b**), DP3 (**c**) and DP4 (**d**) in grapevine canes at E-L 1 and E-L 4. Asterisks denote statistically significant differences between E-L 4 and E-L 1 (Mean values ± SD, *n* = 3, *P ≤ 0.05). Stilbenoids chemical structure can be found in Supplementary Fig. S4

Ten stilbenoids were identified and quantified by UPLC-MS in woody tissues at E-L 1 and E-L 4 (Fig. 5; chemical structures can be found in Supplementary Fig. S4). The content of monomeric stilbenoids like *E*-resveratrol significantly increased 6.3-fold from E-L 1 to E-L 4 (69.1 to 436.5 µg g DW^−1^) while *E*-piceatannol content increased 12.3-fold (9.2 to 113.18 µg g DW^−1^) in the same period (Fig. 5a). Dimeric stilbenoids were more abundant than monomeric ones at both developmental stages, but only δ-viniferin, which was not found at E-L 1, abruptly accumulated at bud burst up to 1.5 mg g DW^−1^. No changes were identified in the amounts of trimeric (Fig. 5c) and tetrameric stilbenoids following bud burst (Fig. 5d).

Despite the observed changes in the metabolomic profile of grapevine woody tissues following bud burst, the amount of total phenolics, measured by the Folin–Ciocalteau method, was not significantly modified. Nonetheless, the antioxidant capacity of the methanolic extracts increased by 48% from 14.0 at E-L 1 to 20.7 mg quercetin eq. g DW^−1^ at E-L 4 (Supplementary Fig. S5), most likely reflecting the above-referred qualitative changes.

### Quantification of aromatic amino acids in grapevine canes

**Fig. 6:**
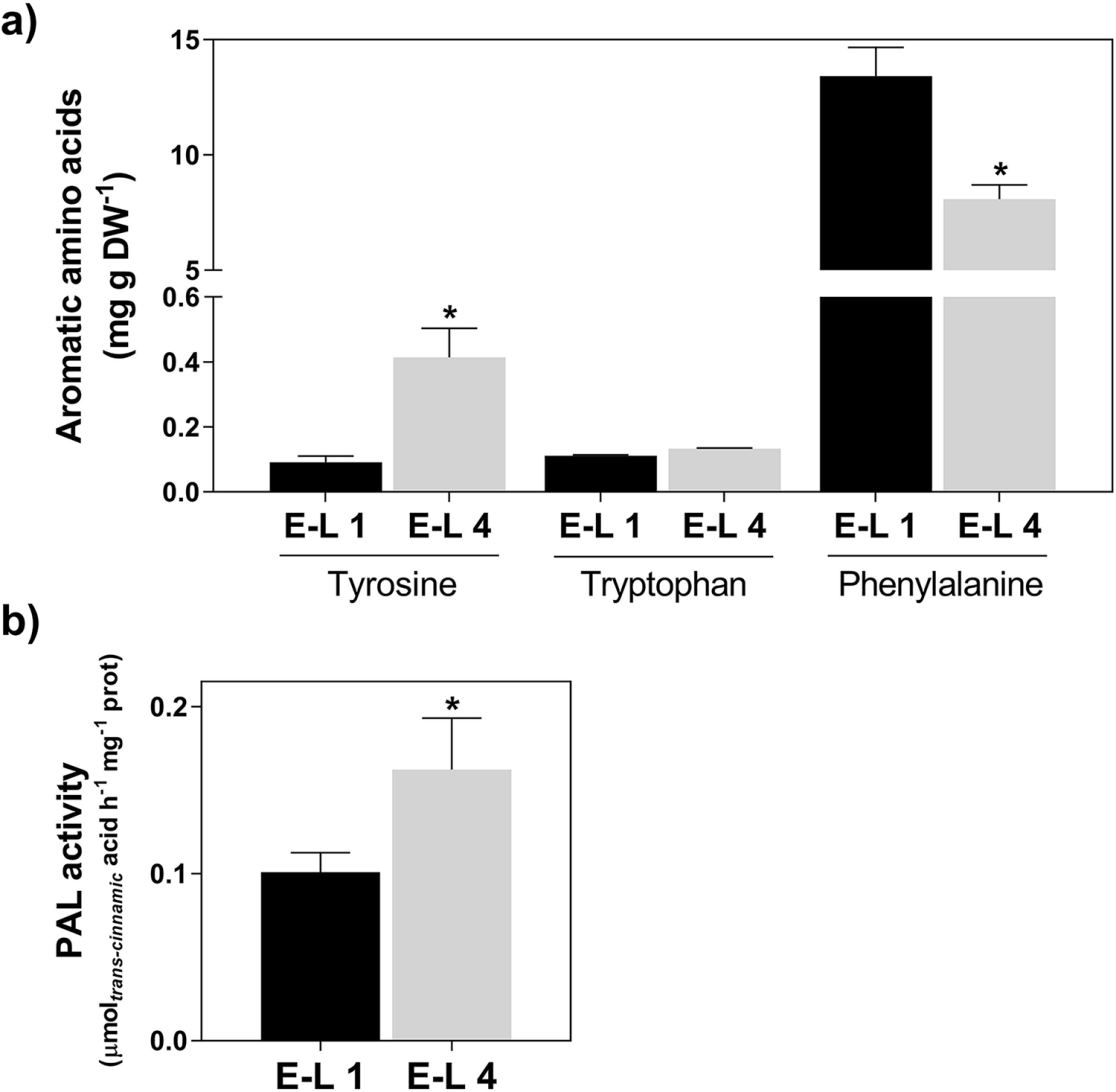
Metabolomic analysis of aromatic amino acids (**a**) and PAL biochemical activity (**b**) in grapevine canes at E-L 1 and E-L 4. Asterisks denote statistically significant differences between E-L 4 and E-L 1 (mean values ± SD, *n* = 3, *P ≤ 0.05)

The content of aromatic amino acids in grapevine woody tissues below the bud determined by UPLC-MS showed that phenylalanine, which accumulates to 13.4 mg g DW^−1^, is by far more abundant (up to150-fold) than tyrosine or tryptophan (Fig. 6a). Following bud burst, while the contents in phenylalanine decreased by 40%, tyrosine levels increased by 450% (from 0.09 at E-L 1 to 0.41 mg g DW^−1^ at E-L 4) and tryptophan did not change. In parallel, in crude protein extracts of the woody canes, the biochemical activity of phenylalanine ammonia-lyase (PAL) increased by 60% (from 0.10 at E-L 1 to 0.16 µmol trans-cinnamic acid h^−1^ mg^−1^ protein at E-L 4) during the transition from dormancy to bud burst (Fig. 6b), which most likely accounted for the observed decrease in phenylalanine levels.

## Discussion

### Bud burst stimulates the shikimate and phenylpropanoid pathways in grapevine woody tissues

In this work, we explored the expression profiles of the phenylpropanoid and shikimate pathways and performed a metabolomic analysis using UPLC-MS in grapevine woody tissues at two developmental stages. Most genes of the shikimate pathway, which controls the synthesis of aromatic amino acids, are stimulated upon bud burst, particularly those encoding the ADT enzymes that catalyze the production of phenylalanine. Similarly, the expression level of genes involved in the transport of l-phenylalanine was also stimulated*.* Moreover, the increase of the enzymatic activity of PAL in woody canes protein extracts from E-L 1 to E-L 4 correlates with previous transcriptomic data showing the regulation of several *VviPAL* genes (Noronha et al. 2021). In agreement, both are consistent with stimulation of the phenylpropanoid pathway following bud burst, which most likely contributes to the decrease in the concentration of its precursor phenylalanine. A similar pattern with an increase in the expression of the *PAL* associated with a higher accumulation level of phenylpropanoids and a decrease in phenylalanine content was already observed in cold stored grape berries (Maoz et al. 2019). These findings strongly support the idea that, following bud burst, plastids located in grapevine woody tissues can de novo synthesize aromatic amino acids which are subsequently exported to the cytosol to fuel the phenylpropanoid pathway.

The stimulation of the phenylpropanoid pathway did not lead to a significant change in the total amount of phenolics but resulted in an enhanced antioxidant capacity of the woody tissue. This effect was associated with significant changes in the abundance of specific metabolites, including some phenolic acids and stilbenoids (discussed below). Of note, the observed increase in woody canes of ferulic acid at E-L 4, may be related to the increased production of cell wall components at this stage, including hemicelluloses or lignin, when secondary growth in grapevine woody tissues is resumed during spring (Noronha et al. 2021).

### Stilbenoids are synthesized and accumulate following bud burst in grapevine woody tissues

In grapevine, most studies on stilbenoids are focused on berries, due to their relevance to the wine industry, or in leaves, due to their role as phytoalexins following pathogen infection (Billet et al. 2020), but they are also constitutively synthesized and accumulated in lignified grapevine organs (Gatto et al. 2008). In this regard, in the present study we demonstrate that at bud burst de novo synthesis and accumulation of the stilbenoids *E*-resveratrol, *E*-piceatannol, and δ-viniferin occurs in woody tissues close to the bud. Furthermore, the correlation between the accumulation of these compounds and the regulation of genes that control their synthesis suggests that stilbenoid accumulation is primarily controlled at the transcriptional level in woody tissues at bud burst. Consistently, *stilbene synthase* (*STS*) genes were upregulated/derepressed during the transition from E-L 1 to E-L 4. In addition, it has been described that *VviMYB14* and *VviMYB15* are fundamental in regulating stilbene accumulation in grapevine in response to several biotic and abiotic stresses (Höll et al. 2013) and that each of the transcription factors may activate the expression of different *STS* genes (Ciaffi et al. 2019). In the present study, the upregulation of 13 *stilbene synthases* genes (*VviSTS3, -4*, *-7*, *-8*, *-13*, *-14*, *-15*, *-16*, *-17*, *-18*, *-19*, *-20*; see Supplementary Table S2 for details) correlates with the increased expression of *VviMYB14*. Thus, we anticipate that *VviMYB14* might be involved in the induction of the *VviSTS* genes leading to the accumulation of stilbenoids, especially considering that some of them (*VviSTS4*, *-7*, *-15* and *-16*) have been shown to synthesize stilbenes (Hain et al. 1993; Thomsik et al. 1997; Parage et al. 2012). Interestingly, even though no increase in flavonoid content was detected by UPLC-MS, an upregulation of chalcone synthase expression, which competes with stilbene synthase for the same substrates, was also observed, suggesting that other undetected compounds may be synthesized in grapevine canes following bud burst.

### Possible role for increased stilbenoids accumulation in grapevine woody tissues following bud burst

Taken together, the present results suggest that specific secondary compounds, including biologically relevant stilbenoids, are actively synthesized when young woody tissues may require additional protection against stress. Interestingly, besides the accumulation of monomeric stilbenoids (piceatannol and resveratrol) found at E-L 4, the levels of *E-*ε-viniferin also increased. The role of dimeric stilbenoids in grapevine has been shown previously, and viniferin production is considered a good indicator of plant resistance against fungal attacks (Pezet et al. 2004). Also, grapevine cv. Vinhão cultured cells accumulate stilbenoids (including viniferin-type compounds) following their inoculation with *Phaeomoniella chlamydospore* autoclaved extracts, a pathogen associated with esca disease (Lima et al. 2012). Thus, it is likely that the stilbenoids accumulated in grapevine woody tissues promote protection against fungal pathogens following bud burst.

Despite the vast body of reports on stilbenoids, their true subcellular localization remains puzzling. In grapevine, several reports have shown that the STS enzyme is localized in the cell wall (Fornara et al. 2008) together with its product stilbenoids (Bellow et al. 2012). The cell wall is a barrier that fungal pathogens must penetrate to access intracellular components and lignin deposition, as well as other phenolics, is recognized as a defense mechanism against these attacks (Ninkuu et al. 2023). Thus, the synthesis and accumulation of stilbenoids in the cell wall is consistent with its antimicrobial and protective properties and may be an effective way to protect the plant from undesired toxic effects (Fornara et al. 2008). Indeed, overexpressing *STS* genes leads to an increased resistance against pathogens in Arabidopsis (Huang et al. 2016), tobacco (He et al. 2018) and grapevine 41B rootstock (Coutos-Thévenot et al. 2001). Strikingly, it was also shown that the overexpression of an *STS* from *Vitis quinquangularis* in *Arabidopsis thaliana* produces plants that are more tolerant of osmotic stress conditions, possibly via the stimulation of the plant antioxidant machinery (Huang et al. 2016). Thus, it is possible that stilbenoids accumulated in cell walls could help mitigating oxidative stress that occurs during secondary growth.

Besides the scientific dimension of the present study, results also hold biotechnological interest since grapevine woody tissues are currently being used as raw material for the extraction of biologically active phenolic compounds and to obtain extracts with antimicrobial activities (Billet et al. 2018a, b; El Khawand et al. 2020).

### Supplementary Information

Below is the link to the electronic supplementary material.

Supplementary file1 (XLSX 26 KB)

Supplementary file2 (DOCX 782 KB)

## Data Availability

The data that support the findings of this study are available in the European Nucleotide Archive with the ENA study accession PRJEB43358.

## Abbreviations

PAL: Phenylalanine ammonia lyase
STS: Stilbene synthase
