# Supplementary material for: Grapevine woody tissues accumulate stilbenoids following bud burst
Source: Planta. 2023 Nov 14;258(6):118. doi: 10.1007/s00425-023-04270-5 (PMC10645632; doi:10.1007/s00425-023-04270-5)
Supplement: Supplementary file 2 — Supplementary file2 (DOCX 782 KB) [file 425_2023_4270_MOESM2_ESM.docx]

**Supplementary Material**

**Supplementary Table S1** Primers used in this study

|  | Forward primer | Reverse primer | Accession number |
| --- | --- | --- | --- |
| VViGAPDH  (Gainza-Cortés et al. 2012) | TTCCGTGTTCCTACTGTTG | CCTCTGACTCCTCCTTGAT | Vitvi17g01598 |
| VviACT1 | GTGCCTGCCATGTATGTTGCCATTCAG | GCAAGGTCAAGACGAAGGATAGCATGG | Vitvi04g01613 |
| VviDAHPS1  (Zhang et al. 2012) | TGCTGCTGGACTCACAGTT | CATGAGCACATCCAGTTG | Vitvi07g03049 |
| VviDAHPS2  (Zhang et al. 2012) | CACCGGAGGGTATGCTTCTA | ACCAAGGGCTTCATCAACAC | Vitvi18g00436 |
| VviDAHPS3  (Zhang et al. 2012) | TGCTTTGGGTAGGTGAGAGAA | GGTTTGTTGCGAGGGTTTAG | Vitvi02g01749 |
| VviAS | CATCTGGAAGCCGTTTTGAC | CATCAGCGGGAACACTATCA | Vitvi06g00617 |
| VviCM1 | AGCTGCCATCAAAGCACAAGAC | TCTTCAACCCTCTGGTAAGTCAGC | Vitvi04g00594 |
| VviADT1 | TGCCTGGTGTAAGAAAGGATGAGC | GGCCATCAGAAGCGACAATCTGAG | Vitvi10g00088 |
| VviADT2 | GGTGGGAATTGATCAGCTAAGACG | GGCCATGTGAGGCTACATACTGAG | Vitvi12g00380 |
| VviSTS3/4  (Ciaffi et al. 2019) | AGAGAATGGTCCCTTTAACG | AACAATGACTCAATTACAATC | Vitvi16g01455/Vitvi16g01481 |
| VviSTS7/8 | AGGTGAAGGATTGGATTGG | TTACATTAAGACATTGAAGGGT | Vitvi16g01482  Vitvi16g01479 |
| VviSTS14 | ATTCCTACGGTTACAAATTAAGTG | CAAAGAAAGTCTAACAATGACTTG | Vitvi16g01482 |
| VviSTS16/17/18 | TTACAGAGGAGGTGCTAC | GCGATAACAGAATGACAA | Vitvi16g00991 |
| VviSTS20 | GGACCAGGCTTAACCATCG | GACTCCAATTTGATACCGTAGAAC | Vitvi16g01475 |
| VviMYB14 | GGAGAGCCTTGGGTATGG | GCAGGGTGTAGTAATGTCG | Vitvi07g00598 |
| VviMYB15 | GCACTGGCGTCAAGAATG | GTCCATAGGCGAGTTCCG | Vitvi05g01733 |

**Supplementary Fig. S1**


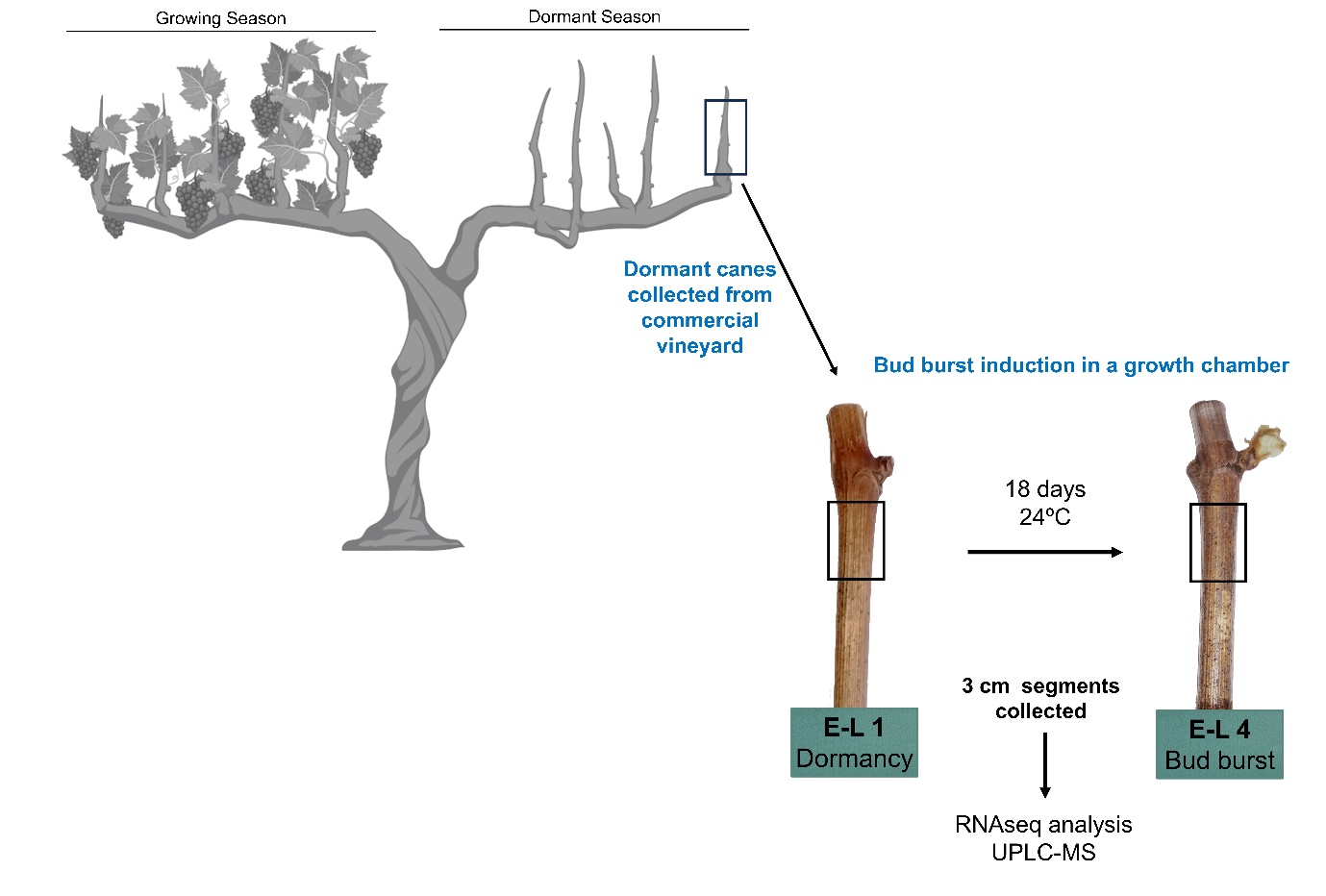


**Supplementary Fig. S1** Experimental design to study stilbenoid metabolism in grapevine cv. Vinhão woody tissues. Dormant canes (E-L 1) were collected from a commercial vineyard and single bud segments of approximately 15 cm were placed in a growth chamber to induce bud burst (E-L 4). 3 cm segments below the bud were collected and used for gene and metabolite analyses.

**Supplementary Fig. S2**

**
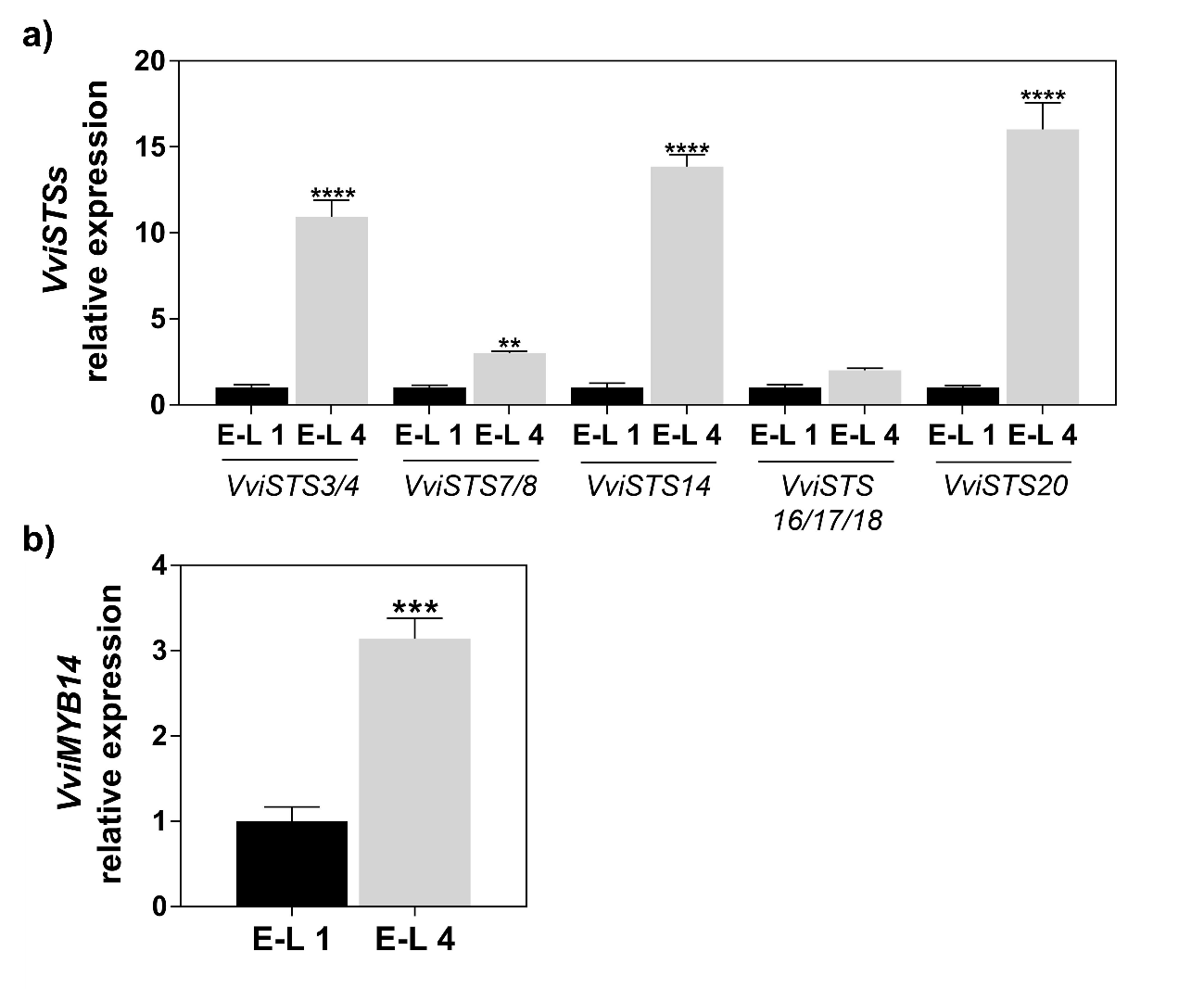
**

**Supplementary Fig. S2** Transcriptional analysis of *VviSTSs* (**a**) and VviMYB14 (**b**) in *Vitis vinifera* cv. Vinhão canes at E-L 1 and E-L 4. Results indicate the mean ± SD of three biological replicates per condition. Asterisks mark significant differences (***P* < 0.01; ****P* ≤ 0.001 *****P* < 0.0001).

**Supplementary Fig. S3**

**
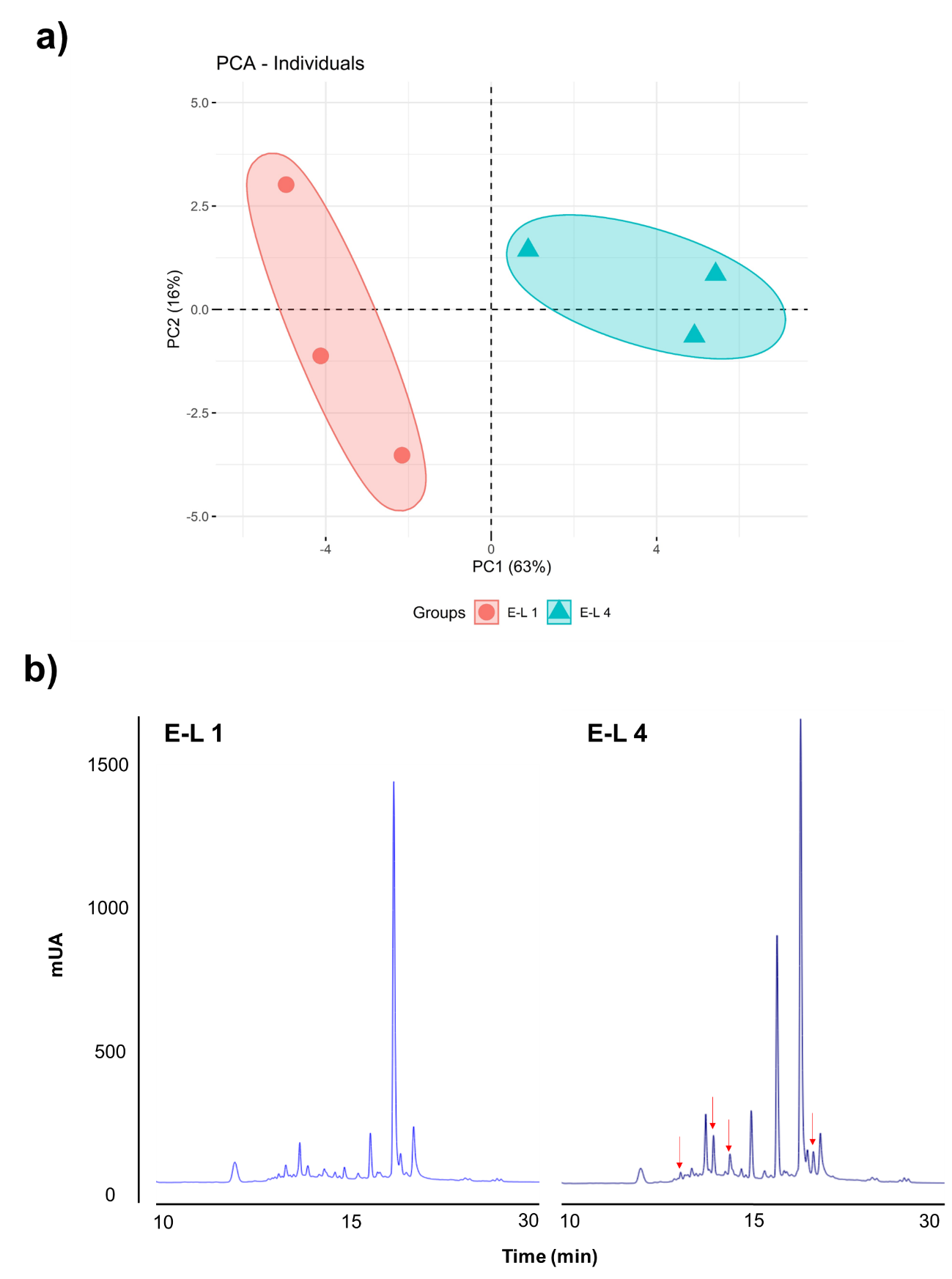
**

**Supplementary Fig. S3** PCA analysis of metabolites detected by HPLC-DAD in methanolic extracts from *Vitis vinifera* cv. Vinhão canes at E-L 1 and E-L 4 (**a**) and their typical chromatograms at 310 nm (**b**). Peaks with clear intensity peaks are marked with red arrows.

**Supplementary Fig. S4**


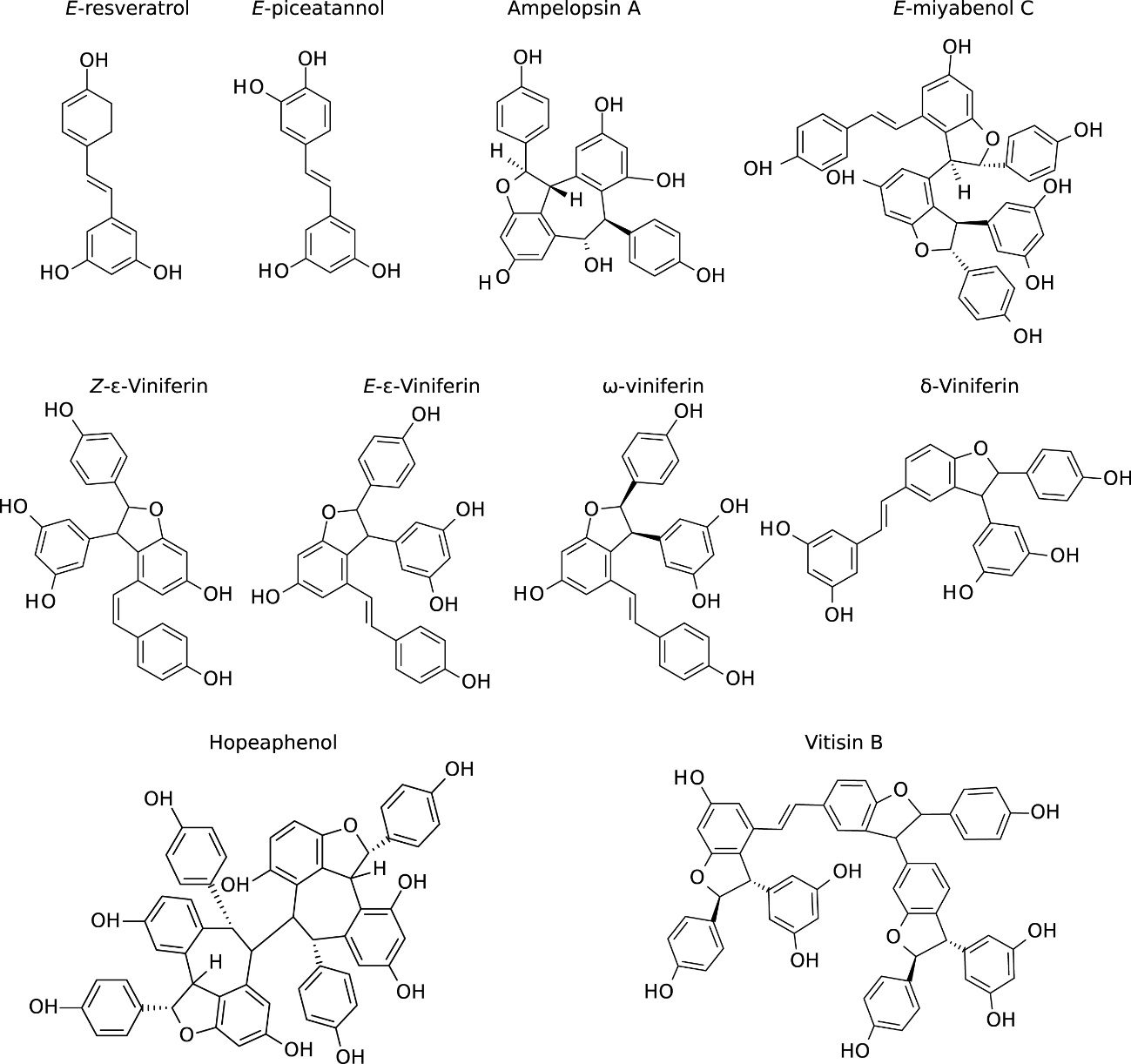


**Supplementary Fig. S4** Chemical structure of stilbenoids quantified in this study.

**Supplementary Fig. S5**

**
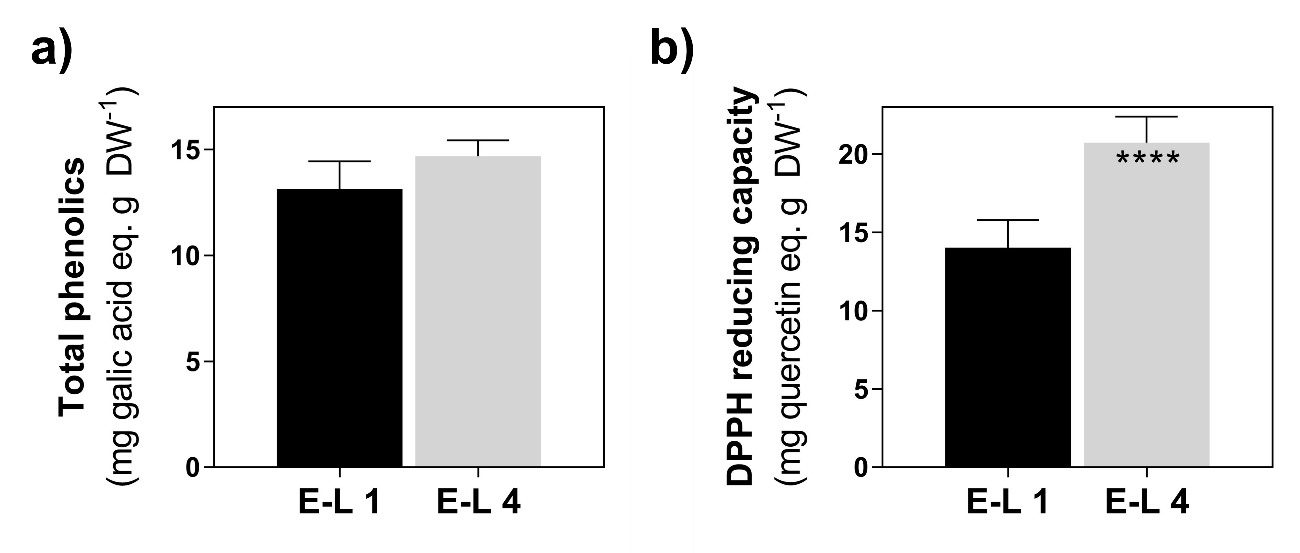
**

**Supplementary Fig. S5** Total phenolics (**a**) and antioxidant capacity (**b**) of methanolic extracts from *Vitis vinifera* cv. Vinhão canes at E-L 1 and E-L 4. Results indicate the mean ± SD of three biological replicates per condition. Asterisks mark significant differences (*****P* < 0.0001).
